# Preoperative planning and safe intraoperative placement of iliosacral screws under fluoroscopic control

**DOI:** 10.1007/s00064-019-0612-x

**Published:** 2019-06-03

**Authors:** Dietmar Krappinger, Richard A. Lindtner, Stefan Benedikt

**Affiliations:** Department of Trauma Surgery, Medical University of Innsbruck, Anichstr. 35, 6020 Innsbruck, Austria

**Keywords:** Posterior pelvic ring injury, Sacral fracture, Sacral dysmorphism, Osseous corridor, Multiplanar reformation, Fluoroscopy, Hintere Beckenringverletzung, Sakrumfraktur, Sakraler Dysmorphismus, Knöcherner Korridor, Multiplanare Reformation, Röntgendurchleuchtung

## Abstract

**Objective:**

Preoperative planning of the starting point and safe trajectory for iliosacral screw (SI screw) fixation using CT scans for safe and accurate fluoroscopically controlled percutaneous SI screw placement.

**Indications:**

Transalar and transforaminal sacral fractures. SI joint disruptions and fracture-dislocations. Non- or minimally displaced spinopelvic dissociation injuries.

**Contraindications:**

Transiliac instabilities. Sacral fractures with neurological impairment requiring decompression. Relevant residual displacement after closed reduction attempts. Insufficient fluoroscopic visualization of the anatomical landmarks of the upper sacrum.

**Surgical technique:**

Preoperative planning of the starting point and the safe screw trajectory using CT scans and two-dimensional multiplanar reformation tools. Fluoroscopically guided identification of the starting point using the lateral view according to preoperative planning. Advancing the guidewire under fluoroscopic control using inlet and outlet views according to the planned trajectory. Predrilling and placement of 6.5 mm cannulated screws.

**Postoperative management:**

Weightbearing as tolerated using crutches. Immediate CT scan in case of postoperative neurological impairment. Generally no screw removal.

**Results:**

Fifty-nine screws were placed in 34 patients using the described technique. There were 2 cases of screw malpositioning (anatomical landmarks inadequately identified and fluoroscopically controlled SI screw fixation should thus not have been performed at all; in a case with sacral dysmorphism, preoperative planning suggested a posterior and/or caudal S1 starting point, respectively, but intraoperatively, selection of a different starting point and screw trajectory resulted in screw malpositioning with iatrogenic L5 nerve palsy).

## Zusammenfassung

**Operationsziel:**

Präoperative Analyse des Eintrittspunkts und des sicheren Korridors für die sakroiliakale (SI) Verschraubung zur sicheren bildwandlergestützten perkutanen SI-Schraubenplatzierung.

**Indikationen:**

Transalare und transforaminelle Sakrumfrakturen. SI-Gelenksprengungen und -Luxationsfrakturen. Nicht- bzw. und minimal dislozierte spinopelvine Dissoziationen.

**Kontraindikationen:**

Transiliakale Instabilitäten. Trümmerfrakturen des Kreuzbeins und Frakturen mit neurologischen Defiziten, die eine direkte Dekompression erfordern. Unzureichende Frakturreposition nach gedeckter Reposition. Unzureichende Darstellung der anatomischen Strukturen im Bildwandler.

**Operationstechnik:**

Präoperative Analyse des Eintrittspunkts und des sicheren Korridors unter Verwendung von Computertomographie-(CT-)Bildern und multiplanarer Reformationstools. Bilderwandlergestütztes Aufsuchen des korrekten Eintrittspunkts in der lateralen Projektion. Vorantreiben des Führungsdrahts in das Kreuzbein unter Kontrolle in der Inlet- und Outlet-Projektion entsprechend der präoperativen Planung. Aufbohren und Einbringen von kanülierten 6,5-mm-Schrauben.

**Weiterbehandlung:**

Schmerzabhängige Vollbelastung mit Stützkrücken. Sofortige postoperative CT-Abklärung bei neu aufgetretenen neurologischen Defiziten. Grundsätzlich keine Schraubenentfernung.

**Ergebnisse:**

Insgesamt 59 SI-Schrauben wurden in 34 Patienten nach beschriebener Methode eingebracht. In 2 Fällen traten Schraubenfehlpositionierungen auf (die anatomischen Strukturen im Bildwandler wurden nicht ausreichend dargestellt, sodass die bildwandlergestützte Verschraubung nicht hätte durchgeführt werden dürfen; bei einem Fall mit sakralem Dysmorphismus ergab die präoperative Analyse einen dorsalen bzw. kaudalen S1-Eintrittspunkt, intraoperativ führte aber die Wahl eines anderen Eintrittspunkts und Schraubenkorridors zu konsekutiver Schraubenfehllage und iatrogener Läsion der L5-Nervenwurzel).

## Introductory remarks

Percutaneous iliosacral screw (SI screw) fixation is a widely used minimally invasive technique for the stabilization of posterior pelvic ring injuries. It is applicable for both high-energy pelvic ring [5, 13, 15, 19] as well as for osteoporotic pelvic fractures [2, 10]. Screw positioning relies on sound intraoperative imaging. Screw malpositioning is one of the major complications of this technique with a relative frequency of up to 17% [14, 20]. Atypical osseous configurations of the upper sacrum, known as “sacral dysmorphism”, represent a considerable risk factor for screw malpositioning [7, 9, 12].

Intraoperative two-dimensional fluoroscopic guidance of SI screw placement using inlet, outlet and lateral views in the standard technique has its limitations in the presence of sacral dysmorphism. For example, indentations of the anterior cortex of S1 (“notch”) are not visible on lateral and inlet projections endangering the L5 nerve root during SI screw insertion [7]. Accordingly, three-dimensional (3D) models of the upper sacrum have been described for the preoperative assessment of the safe osseous screw trajectory [1, 11, 16]. This requires, however, particular and expensive planning software, which is not widely available. Surgical techniques such as computed tomography (CT)-controlled and navigated SI screw fixation have shown to be able to reduce the rate of screw malpositioning [5, 15, 17, 18, 20]. However, again these techniques are not widely available beyond trauma centers due to its high costs and required skills.

Patients sustaining unstable pelvic ring injuries after high-energy trauma are typically transferred to level 1 trauma centers for surgical treatment. Although some of these trauma centers have the facilities for CT-controlled and navigated SI screw fixation, there is still no ubiquitous availability. Moreover, geriatric patients with fragility fractures of the pelvic ring involving the sacral ala are usually treated in peripheral hospitals, which generally have preoperative CT scans and intraoperative fluoroscopy available, while techniques for CT-controlled and navigated SI screw fixation are not provided.

The aim of this article is therefore to present a simple preoperative planning technique to determine the starting point and safe screw trajectory for percutaneous SI screw placement using CT scans and commonly available imaging software tools. Furthermore, it is shown how to transfer the preoperative plan into the intraoperative situation in order to allow for safe and accurate fluoroscopically controlled percutaneous SI screw placement.

## Surgical principles and objective

Safe and accurate percutaneous SI screw fixation under two-dimensional fluoroscopic control even in the presence of sacral dysmorphism via the following two steps:

First step: preoperative planning of the starting point and the safe trajectory for SI screw fixation using native CT scans and commonly available tools.

Second step: Intraoperative fluoroscopically controlled identification of the starting point in the lateral view and advancing the guidewire/screw under fluoroscopic control using inlet and outlet views based on preoperative planning.

## Advantages

- No expensive planning software required
- No intraoperative CT or navigation system required
- Reduced risk of screw malpositioning
- Supine or prone positioning feasible
- Minimally invasive technique with minor iatrogenic soft tissue damage and bleeding risk

## Disadvantages

- Radiation exposure
- Distinct fluoroscopic identification of the anatomic landmarks is mandatory
- Time required for preoperative planning
- Spatial sense and experience in pelvic surgery required for the intraoperative two-dimensional application of the preoperative plan

## Indications

- Transalar and transforaminal sacral fractures after high-energy trauma
- Transalar and transforaminal sacral insufficiency fractures
- SI joint disruptions
- Transiliac and transsacral SI joint fracture dislocations with small bony fragment and thus amenable to SI screw fixation [3]
- Non- or minimally displaced spinopelvic dissociation injuries [13]

## Contraindications

- Transiliac instabilities
- Sacral fractures with neurological impairment requiring surgical decompression
- Relevant residual displacement of the posterior pelvic ring after closed reduction and/or reduction of the anterior pelvic ring
- Insufficient fluoroscopic visualization of the anatomical landmarks of the upper sacrum

## Patient information

- General surgical risks
- Residual risk of screw malpositioning and iatrogenic nerve palsy
- Generally no implant removal

## Preoperative work up

Preoperative planning is the major step for safe fluoroscopically controlled percutaneous SI screw fixation and is therefore described in detail. Pelvic CT scans with a slice depth of 0.6 mm are recommended for preoperative planning. CT scans with slice depths >0.6 mm are also applicable, but may result in inferior image quality during the reformation process. Any imaging software, which supports two-dimensional multiplanar reformation (MPR), is suitable for the preoperative assessment.

### First step—obtaining true inlet and outlet views using the MPR tool

**Fig. 1:**
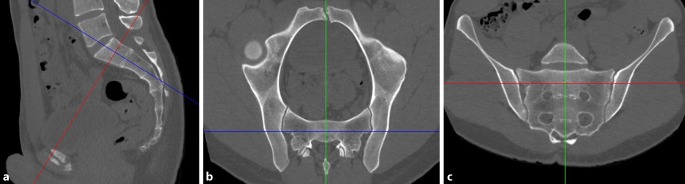
First step of preoperative work-up: obtaining true inlet and outlet views using the multiplanar reformation (MPR) tool. **a** Lateral view, **b** inlet view, **c** outlet view. *Blue line* coronal axis, *red line* horizontal axis, *green line* sagittal axis

In the lateral view, the midsagittal plane is applied (Fig. 1a). The coronal axis (blue line) is adjusted parallel to the posterior border of S1 and the horizontal axis (red line) parallel to the caudal border of S1. The two axes cross in the center of S1.

In the inlet view (Fig. 1b), the sagittal axis (green line) is then adjusted to a true midsagittal plane, which crosses the symphysis anteriorly and the spinous process posteriorly.

In the outlet view (Fig. 1c), the horizontal axis (red line) is now parallel to a line connecting the cranial borders of the SI joint and crosses the center of S1. The midsagittal axis (green line) is parallel to the vertical axis of the sacrum.

### Second step—defining the corridor type and assessing the “notch”

The definition of the corridor type and the assessment of the “notch” is a prerequisite for the determination of the screw trajectory. The corridor type is defined in the inlet view based on the axis of the osseous corridor (white line) in comparison to the coronal axis (blue line).

**Fig. 2:**
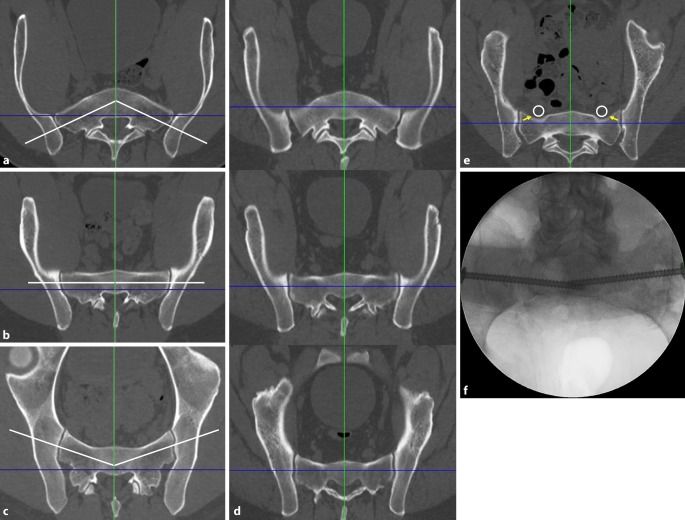
Second step of preoperative work-up: defining the corridor type and assessing the “notch”. **a** Ascending type, **b** horizontal type, **c** descending type, **d** corridor types at different levels of S1 in the same patient, **e** “notch”, **f** intraoperative image. *Blue line* coronal axis, *green line* sagittal axis, *white line* osseous corridor, *white circle* nerve roots L5, *yellow arrows* notch

Ascending type (Fig. 2a): The axis of the osseous corridor runs from lateral posterior to medial anterior. The anterior surface of the sacrum has a convex shape.

Horizontal type (Fig. 2b): The axis of the osseous corridor runs parallel to the coronal axis. The anterior surface of the sacrum has a flat shape.

Descending type (Fig. 2c): The axis of the osseous corridor runs from lateral anterior to medial posterior. The anterior surface of the sacrum has a concave shape.

The corridor type may change within a single sacral body along the craniocaudal extension of the sacrum. Accordingly, the corridor type is assessed in the upper and the lower third of the sacral body as well. Figure 2d shows a sacrum with an ascending corridor in the upper third, a horizontal corridor in the middle third and a descending corridor in the lower third.

A “notch” (yellow arrow in Fig. 2e) is defined as an indentation of the anterior cortex of the sacral ala between the SI joint and the sagittal midline [7]. A notch results in a reduced anteroposterior diameter of the osseous corridor in this part of the screw trajectory. This increases the risk of screw misplacement, which may lead to iatrogenic nerve lesions due to the close vicinity of the notch and the nerve roots L5 (white circle). An indentation of the anterior cortex of the sacral ala requires a posterior entry point and an oblique screw trajectory.

The notch may not be visible in the two-dimensional intraoperative inlet view due to overlapping of the anterior cortex of S1 and S2. This intraoperative image (Fig. 2f) shows the same patient as in Fig. 2e.

### Third step—assessing transverse screw trajectories

**Fig. 3:**
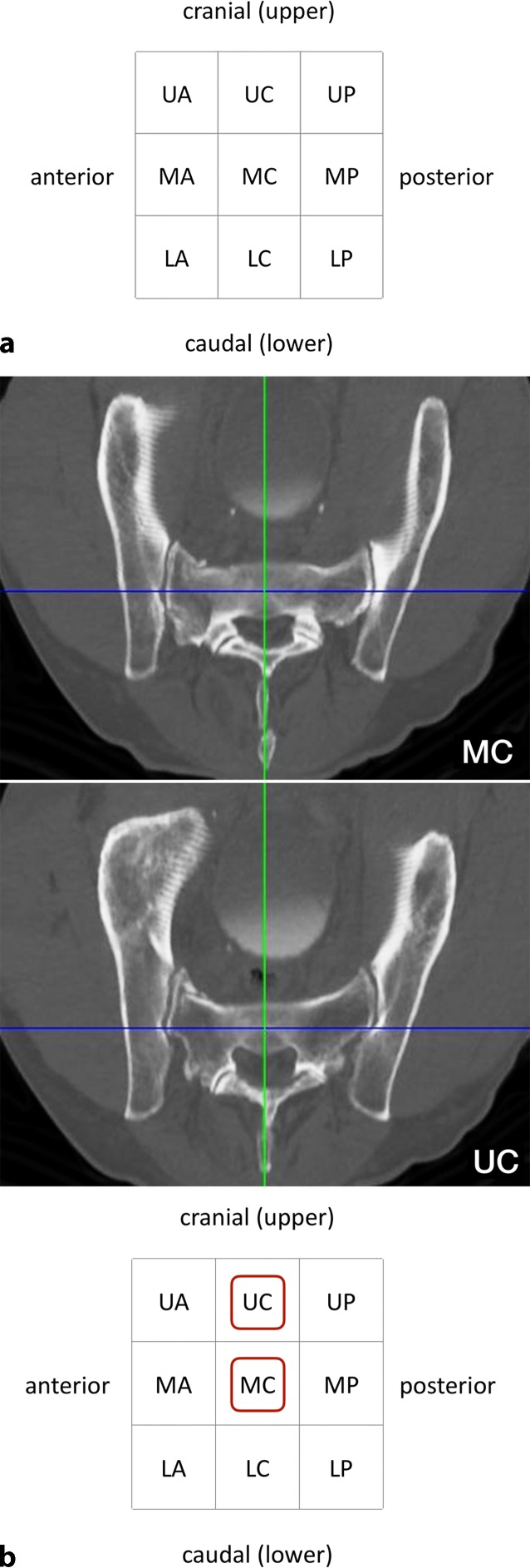
Third step of preoperative work-up: assessing transverse screw trajectories. **a** The lateral view at the level of the S1 body is segmented in a 3 × 3 grid with 9 zones, **b** transverse screw trajectory. *U* upper, *M* middle, *L* lower, *A* anterior, *C* central, *P* posterior, *red circumscribed letters* transverse screw trajectories with safe osseous corridors, *Blue line* coronal axis, *green line* sagittal axis

Intraoperatively, the S1 starting point is identified in the lateral view (Fig. 3a). For preoperative planning using multiplanar CT reconstructions, the lateral view at the level of the S1 body is segmented in a 3 × 3 grid with 9 zones. An **u**pper, **m**iddle and **l**ower third are distinguished from cranial to caudal, which corresponds to the outlet view. An **a**nterior, **c**entral and **p**osterior third are distinguished from anterior to posterior, which corresponds to the inlet view. Accordingly, the zones are labeled as acronyms of two uppercase letters. For example, the “UA” zone corresponds to the upper and anterior third, the “MC” zone to the middle and central third and the “LP” zone to the lower and posterior third.

A transverse screw trajectory (Fig. 3b) following one of the nine zones is preferable over an oblique screw trajectory, which crosses zone borders, for the following two reasons. First, a transverse trajectory allows for the safe use of longer screws or even for the use of transiliac–transsacral screws or bars. Second, the intraoperative two-dimensional fluoroscopic control is much more demanding for oblique trajectories.

Within the transverse screw trajectories, central transverse trajectories (UC, MC and LC) are preferable over anterior and posterior transverse trajectories for the following reason. Small intraoperative deviations from preoperative planning may result in posterior screw misplacement for posterior transverse trajectories (UP, MP and LP) with consecutive iatrogenic lesions of sacral nerve roots in the sacral canal. Small intraoperative deviations from preoperative planning may result in anterior screw misplacement for anterior transverse trajectories (UA, MA and LA) with consecutive iatrogenic lesions of lumbar nerve roots. In contrast, central transverse trajectories may compensate for small intraoperative deviations.

The assessment of the MC transverse trajectory is the first choice due to its central location. This trajectory is therefore also called “Bullseye trajectory”. In the present case there is an adequate osseous corridor for an MC trajectory. There is also an UC trajectory, while the LC trajectory is in close vicinity to the S1 nerve roots. The transverse screw trajectories with safe osseous corridors (MC and UC) are marked in the grid as depicted. Even in the presence of safe transverse trajectories it is advisable to preoperatively also assess safe oblique trajectories as shown below in order to be able to intraoperatively judge whether small intraoperative deviations from the preoperative plan are acceptable.

### Fourth step—assessing oblique screw trajectories

**Fig. 4:**
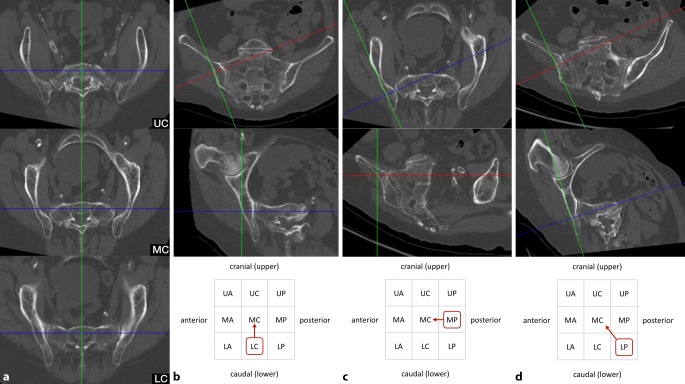
Fourth step of preoperative work-up: assessing oblique screw trajectories. **a** Sacral dysmorphism, **b–d** three main options for oblique S1 screw trajectories. *Blue line* coronal axis, *green line* sagittal axis, *red line* horizontal axis, *red circumscribed letters* represent the zone for screw starting point, *red arrows* indicate the direction of the oblique screw trajectory

The term “sacral dysmorphism” is widely used and several radiographic characteristics of dysmorphic sacral anatomy have been defined [12]. Nevertheless, a clear and generally accepted definition is still lacking. In our opinion, the lack of safe osseous corridors for transverse screw trajectories in S1 may be a reasonable definition of “sacral dysmorphism”. Accordingly, this applies especially for patients with ascending corridors and notches. In the present case of an ascending type (Fig. 4a) there is no osseous corridor for transverse trajectories in the upper (UC), middle (MC) and lower third (LC).

However, there are obviously numerous options for oblique trajectories in S1 in the presence of sacral dysmorphism. Safe oblique trajectories typically run from posterior to anterior in the inlet view and from caudal to cranial in the outlet view. It is advisable to assess an oblique trajectory, which runs oblique in a single plane only and transverse in the orthogonal plane, as this facilitates intraoperative two-dimensional fluoroscopic control. In some cases, however, oblique trajectories in both the inlet and outlet view are inevitable. Accordingly, there are three main options for oblique S1 screw trajectories as shown in Fig. 4b–d:

#### First option (Fig. 4b).

In the outlet view, the intersection of the sagittal axis (green line) and the horizontal axis (red line) is moved to an optional starting point in the lower third on the lateral cortex of the ilium. The horizontal axis is then rotated cranially to reach the middle third in the midsagittal plane. Without any further adjustments in the inlet view, both views now show a safe osseous corridor. The screw trajectory is oblique in the outlet view (“outlet-oblique”), but transverse in the inlet view (“inlet-transverse”) and runs from LC to MC.

#### Second option (Fig. 4c).

In the inlet view, the intersection of the sagittal axis (green line) and the coronal axis (blue line) is moved to an optional starting point in the posterior third on the lateral cortex of the ilium. The coronal axis is then rotated anteriorly to reach the central third in the midsagittal plane. Without any further adjustments in the outlet view, both views now show a safe osseous corridor. The screw trajectory is oblique in the inlet view (“inlet-oblique”), but transverse in the outlet view (“outlet-transverse”) and runs from MP to MC.

#### Third option (Fig. 4d).

This option is required in the presence of sacral dysmorphism and no safe corridor with an oblique trajectory in a single plane. The horizontal axis (red line) is adjusted in the outlet view and the coronal axis (blue line) is adjusted in the inlet view. The screw trajectory is oblique in the inlet view (“inlet-oblique”) and oblique in the outlet view (“outlet-oblique”) and runs from LP to MC.

## Instruments and implants

- Cannulated large fragment cancellous screws (6.5 or 7.3 mm thread diameter)
- Guide wire (2.8 mm diameter)
- Partially threaded screws (16 mm or 32 mm thread) for the application as lag screws (SI joint disruptions and simple sacral fractures without foraminal comminution)
- Fully threaded screws for the application as position screws (comminuted sacral fractures and insufficiency fractures)

## Anesthesia and positioning

- General anesthesia
- Supine or prone positioning
- Prone positioning is advisable for bilateral SI screw fixation
- Supine positioning with a pad or pillow supporting the lumbosacral region

## Surgical technique

(Figs. 5, 6, 7)

**Fig. 5:**
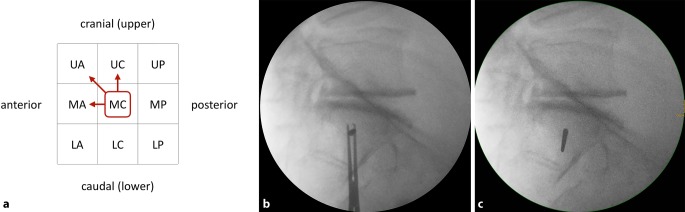
Assessment of the starting point in the lateral view. Three views are mandatory for fluoroscopically controlled SI screw fixation: lateral view, inlet view and outlet view. The techniques for obtaining correct lateral, inlet and outlet views have been previously described in this journal [6]. In addition, the ideal angles for intraoperative fluoroscopic inlet and outlet views can be estimated from preoperative midsagittal computed tomography (CT) reconstructions [4]. It has to be kept in mind that ideal fluoroscopic inlet and outlet view angles are not orthogonal to each other and that the arc of angulation between both views rather averages about 67° [8]. **a** 55-year-old man with a minimally displaced transforaminal sacral fracture after a fall from height. The preoperative assessment shows no signs of sacral dysmorphism with a safe transverse MC corridor (i. e. both the starting point and the endpoint are located in the MC zone in the lateral view). There is also a safe inlet-oblique corridor (MC—MA), a safe outlet-oblique corridor (MC—UC) and an inlet-outlet-oblique corridor (MC—UA). It is planned to perform an SI screw fixation via a transverse MC corridor. **b** In the lateral view a central MC starting point is chosen for a planned strict transverse screw trajectory (MC). **c** The guide wire is advanced to the SI joint for a secure wire purchase in the bone. This additionally allows for assessing the wire trajectory in a lateral view. In the present case fluoroscopic control shows a slight cranial deviation from the planned strict transverse trajectory. Since the outlet-oblique corridor (MC—UC) was preoperatively assessed as safe as well, this trajectory is accepted for further advancement of the wire. *U* upper, *M* middle, *L* lower, *A* anterior, *C* central, *P* posterior

**Fig. 6:**
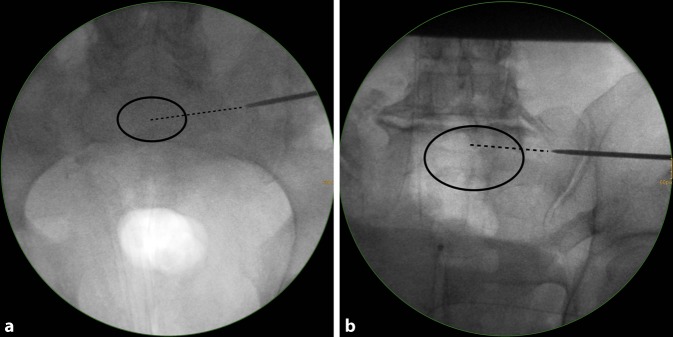
Fluoroscopic control in the inlet and outlet view. The wire trajectory is first controlled in an inlet view (**a**). It targets the central part of S1 (*dotted line*) and is therefore considered as safe. A more anteriorly directed trajectory would have been safe as well according to the preoperative planning (MC—MA). **b** The wire trajectory is then controlled in an outlet view. It follows the MC—UC trajectory, which is safe according to the preoperative planning (MC—UC). *U* upper, *M* middle, *L* lower, *A* anterior, *C* central

**Fig. 7:**
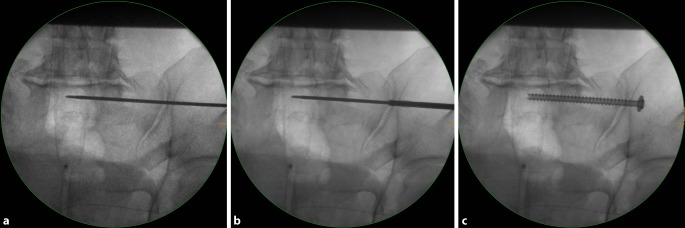
Advancing the guide wire, predrilling and screw implantation. **a** The guide wire is advanced across the sagittal midline in transforaminal sacral fractures. In SI joint disruptions the guidewire should be advanced at least to the extent that the thread (16 or 32 mm) is completely medial of the SI joint in order to obtain a lag screw effect. **b** Predrilling is performed across the SI joint for a total of three cortices. **c** A cannulated 6.5 mm fully threaded cancellous screw is inserted over the guide wire as a position screw without compression of the transforaminal sacral fracture

## Postoperative management

- Weight-bearing as tolerated using crutches
- Immediate CT scan in case of postoperative neurological impairment
- Postoperative CT scan for the assessment of screw position
- Generally no implant removal

## Errors, hazards, complications

(Figs. 8, 9)

**Fig. 8:**
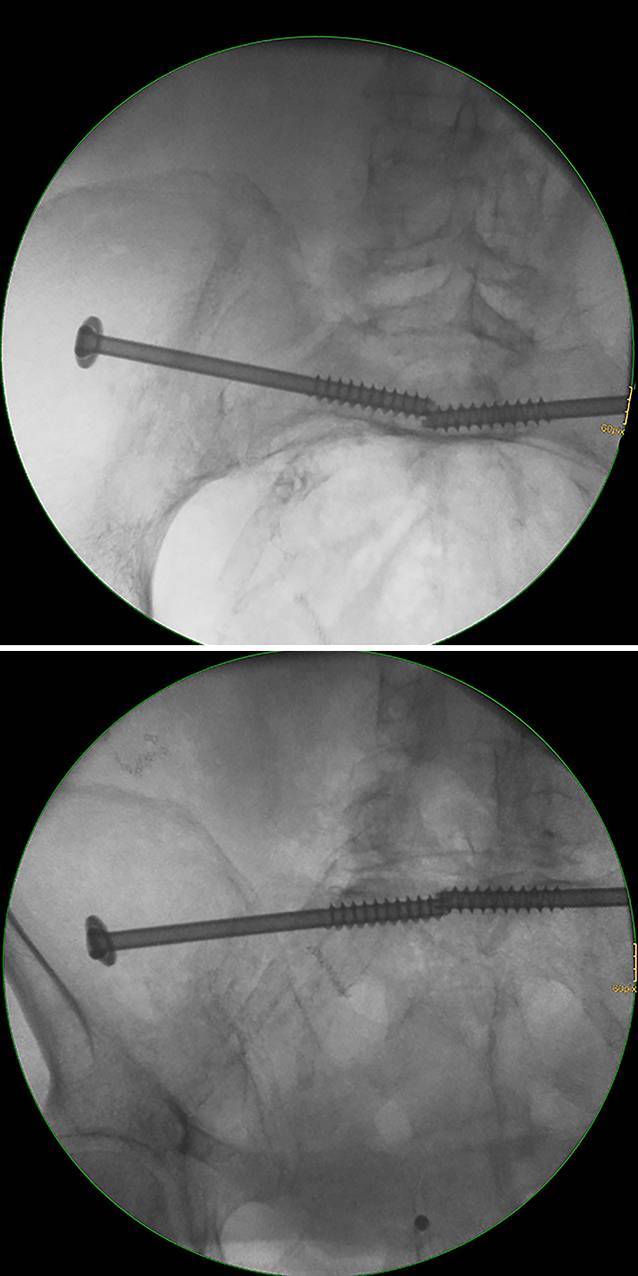
Screw misplacement—intraoperative fluoroscopic control. Fluoroscopically controlled bilateral iliosacral screw fixation in a patient with a U-shaped insufficiency fracture of the sacrum. The screw trajectory follows an inlet-outlet-oblique corridor (MC—UA). The inlet and outlet views show no distinct extraosseous screw trajectory. The same applies for the contralateral side. *U* upper, *M* middle, *A* anterior, *C* central

**Fig. 9:**
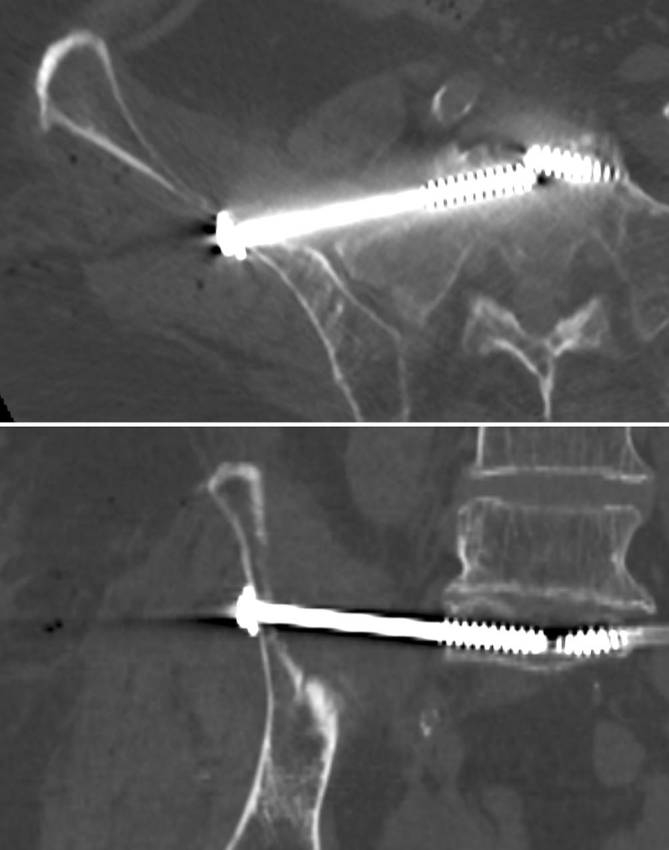
Screw misplacement—postoperative computed tomography (CT) control. Same patient with sacral dysmorphism as in Figs. 4 and 8. The patient showed a foot drop immediately after surgery. The CT control showed a partial extraosseous screw trajectory (“in-out-in”) with lesions of the L5 nerve root

## Results

Osseous corridor types and the presence of a notch were assessed in a series of 1000 CT scans in a recent study using the above described technique [7]. An ascending type was the predominant corridor type in the upper third of S1 (71%), while the rate for ascending types was 21% in the middle third and 5% in the lower third. There were no ascending types in S2. Descending types were the predominant corridor type in the middle and lower third of S1 (66 and 86%, respectively), while the rate was 15% in the upper third. In S2 there were almost exclusively descending types (>95% in all thirds). Notches were present in more than two thirds of the cases in S1 and S2.

Fluoroscopically controlled SI screw fixation using the above-described preoperative planning and insertion technique was performed in 34 patients aged between 22 and 92 years. There were 21 patients with sacral insufficiency fractures and 13 patients with type C injuries of the pelvic ring after high-energy trauma. SI screw fixation was performed bilaterally in all patients with insufficiency fractures and in 4 patients with bilateral posterior pelvic injuries following high-energy trauma, resulting in a total of 59 screws. All surgeries were performed by three pelvic surgeons experienced in fluoroscopically controlled SI screw fixation. Transverse screw trajectories were used for 36 screws and oblique trajectories for 23 screws. There were 2 cases of screw malpositioning. In the first case, fluoroscopic identification of the anatomical landmarks was inadequate and fluoroscopically controlled SI screw fixation should not have been performed. In the second case of a patient with sacral dysmorphism and an ascending type in S1, the preoperative assessment suggested a posterior and/or caudal S1 starting point and an oblique trajectory. Intraoperatively, a different starting point and screw trajectory was chosen resulting in screw malpositioning with iatrogenic L5 nerve palsy (Fig. 9).

Some limitations of this study have to be noted. First, the number of patients is relatively small. Second, postoperative CT scans were performed in the case of postoperative neurological deficits only. Accordingly, besides the two described cases with postoperative neurological deficits we were not able to retrospectively assess the rate of screw malpositioning in patients without neurological deficits. Finally, all SI screw fixations were performed by experienced pelvic surgeons. The rate of neurological complications might therefore be higher for surgeons with less experience.
